# Comparative Impact of Silver Nitrate and Eco-Friendly Silver Nanoparticles on Sexual Behavior, Productivity, and Bioaccumulation in the Reproductive Organs of Japanese Quails

**DOI:** 10.3390/ani15223276

**Published:** 2025-11-13

**Authors:** Hanan Al-Khalaifah, Shabana Naz, Farkhanda Asad, Rifat Ullah Khan, Ala Abudabos, Muhammad Usama, Swaira Ashfaq, Sania Satti, Nudrat Fatima, Hifza Shehzadi, Ibrahim A. Alhidary

**Affiliations:** 1 Environment and Life Sciences Research Center, Kuwait Institute for Scientific Research, Safat 13109, Kuwait; 2 Department of Zoology, Government College University Faisalabad, Faisalabad 38000, Pakistan; farkhanda.asad@gcuf.edu.pk (F.A.);; 3 Physiology Lab, College of Veterinary Sciences, Faculty of Animal Husbandry & Veterinary Sciences, The University of Agriculture, Peshawar 25100, Pakistan; rukhan@aup.edu.pk; 4 Department of Food and Animal Sciences, College of Agriculture, Tennessee State University, Nashville, TN 37209, USA; 5 Department of Animal Production, College of Food and Agriculture Science, King Saud University, Riyadh 11451, Saudi Arabia

**Keywords:** silver nanoparticles, silver nitrate, Japanese quail, reproductive performance, bioaccumulation

## Abstract

Silver nanoparticles (Ag-NPs) are emerging as natural antimicrobial growth promoters in poultry, known for enhancing gut health, immunity, and productivity. This study assessed the effects of Ag-NPs and silver nitrate (AgNO_3_) on sexual behavior, productivity, and silver bioaccumulation in the reproductive tissues of Japanese quails (*Coturnix japonica*). A total of 600 quails (8 weeks old) were randomly assigned to five groups (120 birds/group), each with six replicates (sex ratio 5 male: 15 females). Group 1 (control) received a basal diet, while Groups 2 and 3 were supplemented with Ag-NPs at 10 mg/kg and 20 mg/kg, respectively. Groups 4 and 5 received AgNO_3_ at the same doses for eight weeks. Quails receiving 10 mg/kg Ag-NPs showed significantly improved male mating behavior, increased egg weight and mass, and enhanced egg quality, resulting in the highest fertility and hatchability performance. Silver accumulation in ovaries and testes increased dose-dependently, with the highest deposition observed in the 20 mg/kg Ag-NPs group. Correlation and regression analyses revealed weak and statistically non-significant relationships between ovarian silver levels and fertility and hatchability outcomes. These findings suggested that supplementation with 10 mg/kg Ag-NPs was a safe and effective feed additive for improving both productive and reproductive performance in Japanese quails.

## Simple Summary

This study evaluated the effects of dietary silver nanoparticles (Ag-NPs) and silver nitrate (AgNO_3_) on reproduction and productivity in Japanese quails. Six hundred quails were divided into five groups and fed diets containing 10 or 20 mg/kg of either Ag-NPs or AgNO_3_ for eight weeks. Birds fed 10 mg/kg Ag-NPs showed improved mating behavior, egg quality, fertility, and hatchability compared with other groups. Silver accumulation in reproductive tissues increased with dose but did not significantly affect fertility. Overall, 10 mg/kg Ag-NPs proved safe and effective for enhancing reproductive and productive performance in quails.

## 1. Introduction

Nanotechnology has swiftly emerged as a transformative field across medicine, agriculture, food science, and environmental management [1,2,3,4,5]. Among its most promising branches is the development of metal-based nanoparticles, especially silver nanoparticles (Ag-NPs), owing to their broad-spectrum antimicrobial activity, exceptionally high surface-area-to-volume ratio, and distinctive nanoscale physicochemical behavior [6,7]. These attributes render Ag-NPs highly effective in biomedical applications such as wound healing, drug delivery, biosensing, and the inhibition of microbial growth [8,9].

In the context of poultry farming, the indiscriminate use of antibiotics as growth promoters has raised serious concerns regarding antimicrobial resistance and the presence of drug residues in poultry products. Consequently, there is increasing interest in natural alternatives [10,11,12]. Among these alternatives, metal-based nanomaterials—particularly silver compounds such as silver nitrate (AgNO_3_) and its nanoparticle form (Ag-NPs)—have gained attention for their potent antimicrobial potential and possible use in poultry health management. This situation has spurred interest in natural, non-antibiotic alternatives that support animal health and improve productivity. One such alternative green synthesized Ag-NPs poses great promise due to its ability to deliver antimicrobial effects alongside immune support [7]. These nanoparticles exert their benefits by disrupting microbial cell membranes, generating reactive oxygen species, and inhibiting microbial replication, thereby reducing disease without fostering resistance [6,7,8,9].

Conventional methods for producing Ag-NPs typically involve physical and chemical processes that require high temperatures, toxic reagents, and specialized equipment raising significant environmental and health concerns [12]. In contrast, green synthesis particularly using plant extracts offers an eco-friendly, safe, and cost-effective alternative that adheres to green chemistry principles [13,14,15]. In this method, phytochemicals such as flavonoids, terpenoids, saponins, and phenolic compounds serve as natural reducing and stabilizing agents, converting silver ions (Ag^+^) to metallic silver (Ag^0^) and simultaneously capping the particles to improve stability and functionality [16].

A range of plant extracts have been successfully used to synthesize Ag-NPs, underscoring the method’s versatility and efficacy [16,17]. These biosynthesized nanoparticles often exhibit stronger antimicrobial, antioxidant, anti-inflammatory, and even anticancer activities, enhancing their value for both medical and veterinary applications [15,16,17]. Although both Ag-NPs and AgNO_3_ act by releasing antimicrobial Ag^+^ ions, Ag-NPs offer superior and more sustained efficacy at lower concentrations [18]. Studies show that biosynthesized Ag-NPs achieve larger inhibition zones and lower minimum inhibitory concentrations (MICs) against pathogens such as *S. aureus* and *P. aeruginosa*, compared to equivalent doses of AgNO_3_ [19]. Mechanistically, Ag-NPs not only serve as reservoirs for controlled Ag^+^ release but also directly disrupt microbial membranes [6,7,8,9]. In contrast, AgNO_3_ relies solely on ionic silver and typically requires higher doses to reach similar antimicrobial effects [20]. Additionally, Ag-NPs—especially those under 50 nm—have been shown to penetrate biofilms more effectively and exhibit lower acute toxicity than silver salts (e.g., no tissue staining or excessive ion release), making them a safer and more practical choice [19,20,21].

Building on this, the Japanese quail (*Coturnix coturnix japonica*) serves as an invaluable avian model due to its rapid growth, short reproductive cycle, and economic relevance in meat and egg production [21]. It is thus ideally suited for evaluating the physiological, hematological, and histopathological effects of new feed additives, including nanomaterials [22]. Studies have shown that Ag-NPs can influence parameters such as body weight, feed conversion efficiency, antioxidant enzyme activity, blood chemistry, and tissue integrity in poultry, though findings have been inconsistent likely due to variations in synthesis methods, particle size, and dosage [23]. Therefore, the present study was designed with a clear hypothesis that dietary supplementation with silver nanoparticles (Ag-NPs) and their ionic counterpart, silver nitrate (AgNO_3_), would differentially influence the sexual behavior, productive performance, and tissue silver accumulation in Japanese quails. It was further hypothesized that green-synthesized Ag-NPs, owing to their nanoscale properties, would exert beneficial effects at lower, safer inclusion levels compared to AgNO_3_.

## 2. Materials and Methods

### 2.1. Green Synthesis of Silver Nanoparticles

Leaves of *Azadirachta indica* were collected from Government College University, Faisalabad. They were shade-dried and ground into a fine powder. Two hundred grams of this powder were soaked in 1 L of ethanol (70%) for three days. The solution was filtered through Whatman No. 1 paper and concentrated via rotary evaporation to yield a thick ethanolic extract [24]. This extract was then mixed with a 3 mM silver nitrate (AgNO_3_) solution in a 1:5 ratio and incubated in the dark at room temperature for 24 h to facilitate the formation of silver nanoparticles (Ag-NPs). The formation of Ag-NPs was confirmed immediately by a visible color change from light yellow to brown indicating surface plasmon resonance. UV-Vis spectroscopy (300–700 nm) showed a characteristic peak around 430–450 nm, matching literature reports for neem-based Ag-NPs (peak at ~429 nm in extract). To determine shape and phase, X-ray diffraction (XRD) was performed over a 2θ range of 20–80° at 0.02° steps and 2°/min scan speed. Fourier-transform infrared (FTIR) spectroscopy was used to identify functional groups involved in nanoparticle reduction and stabilization. Dried Ag-NP powder was mixed with KBr, pressed into pellets, and scanned from 4000 to 400 cm^−1^. Key absorption bands at 3292–3311 cm^−1^ (O–H), 1635–1640 cm^−1^ (C=O, N–H), 1040–1049 cm^−1^ (C–O) and a distinct metal–O peak around 591 cm^−1^ confirmed the presence of phenolics, terpenoids, proteins, and flavonoids acting as capping agents.

### 2.2. Experimental Birds and Diet

A total of 600 quails, each 8 weeks old and with similar body weights (190 g ± 16) and egg production rate (50% ± 2), were purchased from the Avian Research and Training Center (ART Centre) at the University of Veterinary & Animal Sciences (UVAS), Lahore. The birds were acclimatized for 10 days in wire cages (each 60 × 40 × 25 cm) under standard hygienic conditions, with the temperature maintained between 20 and 25 °C and 70% humidity. Feed and water were provided ad libitum. The birds were subjected to a 16 h light cycle followed by an 8 h dark cycle. All groups were maintained under identical management practices concerning feeding, lighting, and watering. The quails were divided into five groups, each consisting of 120 birds, further subdivided into six replicates, with 20 birds per replicate (5 males and 15 females, sex ratio 1:3). The first group served as the control and received the basal diet (Table 1). Groups 2 and 3 were supplemented with silver nanoparticles (Ag-NPs) at concentrations of 10 mg/kg and 20 mg/kg of body weight, respectively. Groups 4 and 5 were fed with silver nitrate (AgNO_3_) at the same concentrations (10 mg/kg and 20 mg/kg). The experimental treatments were thoroughly mixed in the drinking water and provided to all 600 quails daily, ensuring uniform intake throughout the 8-week trial period.

### 2.3. Sexual Behavior

Sexual behaviors in Japanese quail were systematically observed and documented over a three-week period, from the tenth to the thirteenth week of life an age range chosen because Japanese quail typically reach sexual maturity around 7–8 weeks, with full reproductive development established by week 10. Behavioral observations were conducted through continuous visual monitoring for a total of one hour per day, on three nonconsecutive days each week. Each observation day included two potential time blocks—morning (06:00–12:00) and afternoon (12:00–18:00)—from which one-hour period was randomly selected according to a fixed rotation schedule (e.g., Day 1: 06:00–07:00, Day 2: 10:00–11:00, Day 3: 14:00–15:00, and so on) to ensure balanced representation of both time periods across weeks. Each one-hour observation was divided into 5 min intervals, during which all birds were scanned and every instance of sexual behavior was recorded. This sampling strategy ensured consistent and representative behavioral data while minimizing observer bias. The following male sexual behaviors were recorded and categorized:

Waltzing: Male circles the female, lowering the wing opposite her.

Wing flapping: Male raises and flaps his wings rapidly above his back.

Tidbitting: Male simulates finding food to attract the female.

Rear approach: Male grasps the female’s neck feathers with his beak.

Mounting: Male climbs onto the female’s back and grips her wing feathers with his feet.

Treading and full copulation: Male performs rhythmic treading followed by cloacal contact, indicating successful mating [25].

### 2.4. Estimation of Productive Performance

Each day, the number of eggs laid was recorded, and each egg was marked upon collection. Freshly laid eggs were immediately weighed using an electric egg scale. At the end of the experiment, the total egg mass was calculated. All eggs laid during the final week of the trial were collected and stored at 12 °C for further analysis.

Hen-day egg production (HDEP) was calculated using the following formula [26]:

$$\mathrm{HDEP}\left(\%\right)=\frac{\left(\mathrm{Total}\;\mathrm{eggs}\;\mathrm{laid}\;\mathrm{during}\;\mathrm{the}\;\mathrm{period}\right)}{\mathrm{Total}\;\mathrm{live}\;\mathrm{hens}}\times 100$$

Egg mass (g/hen/day) was calculated using:

$$\mathrm{Egg}\;\mathrm{mass}=\frac{\mathrm{HDEP}\;\left(\%\right)\;\times \;\mathrm{Average}\;\mathrm{egg}\;\mathrm{weight}\;\left(g\right)}{100}$$

### 2.5. Estimation of Egg Quality

Sixty eggs (ten per replicate) were collected from each experimental group during the 10th and 12th weeks for egg-quality analysis. Each egg was labeled on the shell with its collection date and replicate number using food-grade ink, ensuring accurate traceability. Eggs were weighed with an electronic balance accurate to 0.01 g. Length and width were measured using a micrometer, averaging three readings from different points. Albumen height was determined using the same micrometer, and albumen diameter was measured with slide calipers [27].

The following formulas were used to assess egg quality:

*Shell Weight* (g)

Shell weight was measured directly using a digital weighing balance.

*Shell Thickness* (mm)

Shell thickness was measured using a micrometer at three points (broad end, narrow end, and equator), and the average value was recorded.

*Albumen Weight* (g)

Albumen Weight = Egg Weight − (Shell Weight + Yolk Weight)

*Yolk Weight* (g)

Yolk weight was measured directly after separating the yolk from albumen.

*Haugh Unit*

Haugh Unit = 100 × log_10_ [Albumen Height (mm) + 7.57 − 1.7 × (Egg Weight)^0.37]

*Shape Index* (%)

Shape Index = (Egg Width/Egg Length) × 100

*Yolk Index* (%)

Yolk Index = (Yolk Height/Yolk Diameter) × 100

### 2.6. Estimation of Reproductive Performance

A total of 600 eggs per treatment group (100 per replicate) were incubated for 17 days. Incubation conditions were maintained at 38.5 °C in the setter phase and 37 °C in the hatcher phase. Relative humidity was held at 65% during the setter phase and 85% during the hatcher period. Fertility and hatchability percentages were calculated using the following Equations [26]:

Fertility (%) = (Number of fertile eggs/Total egg set) × 100

Hatchability (%) = (Number of hatched chicks/Total egg set) × 100

### 2.7. Bioaccumulation in Ovaries and Testes

At the end of the trial, five quails from each replicate were euthanized by cervical dislocation. Their testes and ovaries were dissected and thoroughly rinsed with deionized water to remove all traces of blood. The tissues were then cut into small pieces and kept on ice until further analysis. Sample digestion was carried out using the standard protocol. A 0.5 g dried tissue sample was placed in Teflon tubes and mixed with 6 mL of a 1:1 HClO_4_ and HNO_3_ solution. Samples were shaken vigorously for 12–16 h to initiate digestion. They were then heated in a 70 °C water bath for 30 min and transferred to a hot plate until the solution became clear. After cooling, the digested solution was transferred into a volumetric flask and brought up to volume with deionized distilled water. Silver concentration was analyzed using an atomic absorption spectrometer (Aurora AI 1200, Vancouver, BC, Canada) set at a wavelength of 328.1. nm.

### 2.8. Calculation of Silver Concentration

Ovarian and testicular samples were analyzed for silver accumulation following the method of Sirotkin et al. [28] with minor modifications. Approximately 1 g of each tissue sample was accurately weighed and digested in a mixture of nitric acid (HNO_3_) and perchloric acid (HClO_4_) until a clear solution was obtained. The digested samples were then filtered and diluted to a final volume of 25 mL with deionized water. Silver concentration in the diluted samples was determined using a Flame Atomic Absorption Photometer (Model AA-7000, Shimadzu, Kyoto, Japan).

### 2.9. Statistical Analysis

The impacts of different silver nanoparticle (Ag-NPs) and silver nitrate (AgNO_3_) sources on sexual behavior, reproductive outcomes, and silver bioaccumulation in ovaries and testes were assessed using one-way ANOVA in SPSS (Version 21). Post hoc comparisons of group means were performed with Tukey’s test, considering statistical significance at *p* < 0.05. Additionally, Pearson correlation analysis was employed to explore the relationships between ovarian silver concentrations and fertility, as well as between ovarian silver levels and hatchability rates. Regression analysis was also conducted to quantify the strength and nature of associations between ovarian silver bioaccumulation and reproductive performance, providing insights into predictive relationships among these variables.

## 3. Results

### 3.1. Effects of Ag-NPs and AgNO_3_ on Sexual Behavior

The mean frequency of sexual behavior varied highly significantly (*p* < 0.05) among groups of *C. japonica* (Table 2). The highest waltzing (15.25 ± 0.06), wing flapping (86.22 ± 0.60), tidbitting (3.45 ± 0.06), rear approach (76.28 ± 0.51), mounting (65.31 ± 0.53), and treading and full copulation (85.07 ± 0.62) were observed in birds treated with Ag-NPs low dose, compared to other treatment groups.

### 3.2. Effects of Ag-NPs and AgNO_3_ on Productive Performance

The mean values of egg weight and egg mass varied highly significantly (*p* < 0.05) among the groups of quails, while HDEP showed non-significant differences (*p* > 0.05) (Table 3). The highest egg weight (11.33 ± 0.50) and egg mass (8.65 ± 0.10) were observed in Ag-NPs low dose as compared to AgNO_3_ treated groups.

### 3.3. Effects of Ag-NPs and AgNO_3_ on Egg Quality

The mean values for shell weight, yolk weight, Haugh unit, shape index, and yolk index differed significantly (*p* < 0.05) among the experimental groups of quails, whereas shell thickness and albumen weight did not show significant variation (*p* > 0.05) (Table 4). The Ag-NPs low-dose group exhibited the highest values for shell weight, Haugh unit, and yolk index compared to the other groups.

### 3.4. Effects of Ag-NPs and AgNO_3_ on Reproductive Performance

The mean values of reproductive performance varied highly significantly (*p* < 0.05) among the groups of quails (Table 5). The highest fertility (81.50 ± 0.13) and hatchability (83.28 ± 0.29) percentages were observed in Ag-NPs (10 mg/kg) group as compared to other treatments.

### 3.5. Effects of Ag-NPs and AgNO_3_ on Bioaccumulation in Reproductive Organs

The mean values of bioaccumulation in ovaries and testes varied highly significantly (*p* < 0.05) among the groups of quails (Table 6). The highest bioaccumulation in ovaries (0.97 ± 0.05) and testes (3.38 ± 0.13) was observed in Ag-NPs (20 mg/kg) group as compared to other treatments.

### 3.6. Impact of Ovarian Silver Bioaccumulation on Fertility in Japanese Quails

The regression analysis reveals a very weak and statistically insignificant relationship between ovary bioaccumulation and fertility (Table 7). The Pearson correlation coefficient is 0.136, with a *p*-value of 0.314, indicating no meaningful linear association. The regression model explains only 1.9% of the variance in fertility (R^2^ = 0.019), and the adjusted R^2^ is negative (−0.057), suggesting that the model performs worse than predicting the mean fertility value. The unstandardized coefficient for ovary bioaccumulation is 1.10, but with a *p*-value of 0.628, this effect is not statistically significant.

### 3.7. Impact of Ovarian Silver Bioaccumulation on Hatchability in Japanese Quails

The regression analysis reveals a weak and statistically insignificant relationship between ovarian silver bioaccumulation and hatchability (Table 8). The Pearson correlation coefficient is r = 0.283, with a *p*-value of 0.153, indicating no meaningful linear association. The regression model explains only 8.0% of the variance in hatchability (R^2^ = 0.080), and the adjusted R^2^ is 0.010, suggesting that the model performs worse than predicting the mean hatchability value. The unstandardized coefficient for ovarian bioaccumulation is 4.12, with a *p*-value of 0.306, indicating that this effect is not statistically significant.

## 4. Discussion

The present investigation demonstrated significant improvements in the sexual behavior of Japanese quails following dietary supplementation with Ag-NPs and AgNO_3_. Birds treated with the low-dose Ag-NPs group (10 mg/kg) exhibited the highest frequencies of waltzing, wing flapping, tidbitting, rear approach, mounting, and full copulation. These behavioral enhancements may be attributed to the physiological role of trace elements like silver in modulating endocrine functions, particularly testosterone regulation, which is closely linked with sexual display and mating success in avian species [29]. Previous studies have reported similar stimulatory effects on reproductive behaviors through nanoparticle-mediated hormonal enhancement [30,31]. The bioactive nature and high reactivity of Ag-NPs might have facilitated improved nutrient absorption and hormonal efficiency, resulting in increased sexual vigor compared to AgNO_3_-treated groups, which showed comparatively diminished performance [30,31,32]. However, quails supplemented with AgNO_3_—especially at the higher dose (20 mg/kg)—exhibited a marked decline in sexual behavior parameters compared to both control and Ag-NPs groups. This reduction suggests that ionic silver from AgNO_3_ may exert mild toxic or inhibitory effects on reproductive physiology, possibly through oxidative stress, disruption of Leydig cell function, or altered androgen synthesis. Similar findings have been reported in earlier studies where excessive Ag^+^ exposure interfered with endocrine signaling and sperm motility in avian and mammalian models [32].

Productive performance also responded favorably to Ag--NPs supplementation, particularly in egg-related metrics. Although hen--day egg production (HDEP) did not differ significantly among groups, both egg weight and egg mass were considerably increased in the low-dose Ag–NPs group. This enhancement likely results from several mechanistic pathways working in concert: first, Ag–NPs release Ag^+^ ions and generate reactive oxygen species that suppress pathogenic gut bacteria (e.g., *E. coli*, *Salmonella*) while supporting beneficial microbes (e.g., *Lactobacillus*), thereby enhancing nutrient absorption [33]. Second, improved gut integrity characterized by healthier intestinal villi facilitates the uptake of proteins, lipids, and minerals essential for egg formation [26,33]. Third, Ag–NPs upregulate antioxidant enzymes such as glutathione peroxidase and catalase, which reduce oxidative damage in intestinal tissues and preserve nutrient transport functions [34]. Fourth, by modulating gut immune gene expression (e.g., downregulating TLR2/TLR4 activation), metabolic energy is likely redirected from immune defense toward reproductive processes [33,34,35]. Collectively, these mechanisms—antimicrobial activity, improved nutrient digestion and absorption, oxidative protection, and reduced immune burden—contribute to the observed increase in egg weight and egg mass at 10 mg/kg Ag--NPs [34]. Similar findings have been reported in earlier studies where nano-supplemented trace elements enhanced egg parameters through increased bioavailability and enzymatic activity [36]. Conversely, AgNO_3_ supplementation—particularly at 20 mg/kg—significantly reduced egg weight and egg mass, likely due to the cytotoxic nature of free silver ions that may damage intestinal mucosa, disrupt nutrient transporters, and impair hepatic metabolism. These adverse effects suggest that the ionic form of silver (AgNO_3_) is less physiologically tolerable than its nanoparticulate counterpart, consistent with reports of reduced growth and productivity in birds exposed to AgNO_3_ at comparable doses [32,36].

Marked improvements were noted in both internal and external egg quality traits following administration of Ag–NPs. Birds receiving the low dose (10 mg/kg) exhibited the highest values for shell weight, yolk weight, Haugh unit, shape index, and yolk index traits widely recognized as key indicators of egg freshness, shell strength, and internal composition [37,38]. This notable enhancement likely results from the antioxidant properties of Ag NPs—evidenced by increased activity of glutathione peroxidase (GSH Px), superoxide dismutase (SOD), and catalase, which prevent lipid peroxidation (indicated by lower malondialdehyde levels), thereby improving Haugh unit and yolk index [37,38,39]. This mechanism is well-documented in selenium nanoparticle studies, where enzyme upregulation preserves protein integrity in albumen and yolk membranes [40]. Ag NPs, like selenium nanoparticles, can scavenge reactive oxygen species, preserve protein integrity, and maintain structural proteins essential for shell and albumen strength [37,38,39]. Additionally, Ag–NPs may improve trace element uptake and support metabolic processes vital for egg formation. These combined effects mirror findings from selenium nanoparticle research, where higher Haugh units and shell strength were linked to enhanced antioxidant defenses and trace element absorption [37,38,39,40,41]. In contrast, quails treated with AgNO_3_ showed reduced values for most egg quality parameters, particularly yolk weight, shape index, and Haugh unit. This pattern likely reflects the oxidative and cytotoxic stress induced by soluble Ag^+^ ions from AgNO_3_, which can impair albumen protein synthesis, disrupt calcium metabolism, and weaken eggshell deposition. Therefore, while Ag-NPs promoted egg quality through antioxidant protection, AgNO_3_ appeared to compromise these traits through oxidative and metabolic disruption.

Reproductive parameters, including fertility and hatchability, were significantly enhanced in the Ag–NPs low–dose group. This effect likely reflects a balance between mild bioactive stress and beneficial trace mineral availability; at low doses, silver ions from Ag NPs can modulate hormonal signaling, antioxidant activity, and cellular function [42]. Released Ag^+^ ions exhibit high affinity for thiol groups (–SH) in proteins like glutathione and metallothioneins, temporarily tweaking redox balance and activating endogenous antioxidant defenses rather than overwhelming them [43]. This subtle shift may boost mitochondrial function in gametes—supporting follicular maturation without inducing overt oxidative damage [44]. Additionally, low dose Ag NPs can stimulate mitochondrial-dependent apoptosis in damaged embryonic cells, promoting selective embryo retention and healthier hatchlings through quality control mechanisms in early development [45]. Previous findings with other nano—minerals (e.g., selenium) also suggest that controlled nanoparticle exposure supports ovarian and testicular function, modulates hormone secretion, and protects reproductive tissues from oxidative stress ultimately enhancing fertility and hatchability [40,41]. Conversely, the lower fertility and hatchability observed in AgNO_3_-treated quails indicate that ionic silver exerted reproductive toxicity at both tested levels. Free Ag^+^ ions may have induced oxidative stress, damaged gonadal tissue, and disrupted gametogenesis, leading to impaired fertilization and embryonic development. Similar inhibitory effects of AgNO_3_ on reproductive outcomes have been demonstrated in other avian and mammalian studies, where nitrate-derived silver increased follicular atresia and reduced sperm motility via oxidative and endocrine interference [43,44,45].

Substantial deposition of silver was detected in the ovaries and testes of quails supplemented with Ag–NPs, particularly at the higher dose (20 mg/kg). This pattern suggests efficient bioaccumulation facilitated by the nanoscale size and high surface area of Ag NPs, enabling superior absorption and intracellular retention [46]. Mechanistically, Ag NPs can cross the cellular membrane via endocytosis or disrupt membrane integrity, acting as a ‘Trojan horse’ that enters cells intact and then slowly releases Ag^+^ ions within intracellular compartments—especially lysosomes—leading to elevated local ion concentration, reactive oxygen species generation, and enhanced intracellular retention [46,47,48]. In reproductive tissues, such deposition may enhance steroidogenesis, gamete quality, and overall reproductive function. Previous findings have confirmed that nano—formulations of trace elements result in higher tissue assimilation and better functional outcomes compared to conventional salts [49,50]. However, AgNO_3_ exposure also led to detectable silver residues in reproductive organs, albeit at lower levels than Ag-NPs. This suggests that although both forms of silver can bioaccumulate, the ionic Ag^+^ from AgNO_3_ may distribute more rapidly and be excreted sooner, reducing tissue retention but potentially increasing systemic oxidative stress during circulation. This transient accumulation may contribute to the observed decline in reproductive and productive traits among AgNO_3_-treated quails.

A positive trend was initially anticipated between ovarian bioaccumulation and reproductive success; however, regression analysis revealed no statistically significant linear correlation between ovarian silver levels and fertility or hatchability, with models explaining only a small share of variance. These findings imply that while silver does accumulate in reproductive tissues, its direct influence on fertility and hatchability may not follow a simple linear pattern and could be influenced by a broader set of physiological and environmental interactions [51,52]. Despite accumulation of silver in reproductive tissues, its impact on reproductive outcomes appears mediated through complex biochemical pathways—particularly oxidative stress-induced apoptosis in ovarian follicles and impaired steroidogenesis in granulosa cells via downregulation of enzymes like CYP19A and 3β HSD [53]. Studies in rodents and cattle have shown that silver nanoparticle exposure elevates reactive oxygen species and lipid peroxidation, diminishes antioxidant defenses (e.g., SOD, TAC), increases follicular atresia and caspase mediated apoptosis, and reduces estrogen production through lowered aromatase expression [54]. The present findings thus suggest that while Ag-NPs at low doses exhibit beneficial bioactivity, AgNO_3_ exposure tends toward cytotoxicity and endocrine disruption, underscoring the importance of silver form, dose, and bioavailability in determining reproductive outcomes.

## 5. Conclusions

In conclusion, this study demonstrated that administering Ag-NPs significantly enhanced sexual behavior, productive performance, egg quality, and reproductive traits in Japanese quails compared to AgNO_3_. The low dose of Ag-NPs (10 mg/kg) notably improved mating activity, egg weight, egg mass, and shell and yolk quality, as well as fertility and hatchability. Additionally, silver deposition in both ovaries and testes increased with concentration, highlighting the efficient bioaccumulation of Ag-NPs.

## Figures and Tables

**Table 1:** Major components and nutritional contents of Japanese quail diets.

Ingredients	Contents (%)
Yellow Corn	49.25
Soya bean meal	32.18
Wheat starch	10.15
Limestone	6.5
Di-Calcium Phosphate	1.16
Salt (NaCl)	0.3
Alfalfa leaf powder	0.16
Vitamin and mineral premixture *	0.3
Calculated analysis *	
ME, Kcal/kg	2830
Crude protein	22.63
Crude Fiber	2.21
Ether Extract	2.19
Calcium	2.82
Phosphorous	0.33
Methionine + cysteine	0.72
Methionine	0.44
Lysine	1.01

* Each kg of vitamin and mineral mixture included the following: 10,000 IU of retinol, 3500 IU of cholecalciferol, 35 IU of tocopherol, 1.67 mg of phylloquinone, 1.67 mg of thiamine, 2 mg of riboflavin, 3.67 mg of pyridoxine, 0.012 mg of cyanocobalamin, 6.67 mg of pantothenic acid, 16.7 mg of nicotinic acid, 1.67 mg of folic acid, 0.07 mg of biotin, 400 mg of choline chloride,0.033 mg Selenium, 133.4 g of Mg, 90 mg of Mn, 80 mg of Zn, 25 mg of Fe, 1.67 mg of Cu, and 0.8 mg of I.

**Table 2:** Sexual behavior (Mean ± SD) of *C. japonica* treated with low and high doses of Ag-NPs and AgNO_3_.

Parameter	Control	Ag-NPs (10 mg/kg)	Ag-NPs (20 mg/kg)	AgNO_3_ (10 mg/kg)	AgNO_3_ (20 mg/kg)	*p* Value
Waltzing	8.16 ± 0.04 ^d^	15.25 ± 0.06 ^a^	12.51 ± 0.10 ^b^	10.44 ± 0.05 ^c^	10.15 ± 0.05 ^d^	0.01 ***
Wing flapping	62.26 ± 0.24 ^d^	86.22 ± 0.60 ^a^	80.25 ± 0.38 ^b^	65.27 ± 5.61 ^c^	61.14 ± 0.10 ^cd^	0.01 ***
Tidbiting	1.06 ± 0.02 ^c^	3.45 ± 0.06 ^a^	3.28 ± 0.12 ^b^	1.8 ± 0.05 ^c^	1.15 ± 0.13 ^c^	0.01 ***
Rear approach	58.8 ± 0.09 ^e^	76.28 ± 0.51 ^a^	74.16 ± 0.62 ^b^	60.92 ± 0.13 ^c^	58.31 ± 0.47 ^d^	0.01 ***
Mounting	49.61 ± 0.65 ^d^	65.31 ± 0.53 ^a^	63.73 ± 0.45 ^b^	52.39 ± 0.42 ^c^	51.48 ± 0.30 ^c^	0.01 ***
Treading and full copulation	63.97 ± 0.36 ^d^	85.07 ± 0.62 ^a^	80.91 ± 0.55 ^b^	72.13 ± 0.22 ^c^	69.13 ± 0.42 ^c^	0.01 ***

SD = Standard deviation, Ag-NPs = Silver nanoparticles, AgNO_3_ = Silver nitrate: The means values with distinct superscripts (^a, b, c, d, e^) in a row exhibit a substantial variation at (*p* < 0.05). *** = Highly significant.

**Table 3:** Productive Performance (Mean ± SD) of *C. japonica* treated with low and high doses Ag NPs and AgNO_3._

Parameter	Control	Ag-NPs (10 mg/kg)	Ag-NPs (20 mg/kg)	AgNO_3_ (10 mg/kg)	AgNO_3_ (20 mg/kg)	*p* Value
HDEP	67.67 ± 1.5	71.00 ± 1.00	73.34 ± 1.50	66.34 ± 1.50	61.00 ± 2.00	0.296 ^NS^
Egg weight (g)	11.00 ± 0.5 ^a^	11.33 ± 0.00 ^a^	11.00 ± 0.00 ^a^	9.00 ± 0.00 ^c^	10.00 ± 0.19 ^b^	0.01 ***
Egg mass (g)	8.35 ± 0.10 ^a^	8.65 ± 0.26 ^a^	8.37 ± 0.25 ^a^	7.60 ± 0.30 ^b^	7.70 ± 0.22 ^b^	0.01 ***

SD = Standard deviation, Ag-NPs = Silver nanoparticles, AgNO_3_ = Silver nitrate: The means values with distinct superscripts (^a, b, c^) in a row exhibit a substantial variation at (*p* < 0.05). ^NS^ = Non-Significant, *** = highly significant.

**Table 4:** Egg Quality (Mean ± SD) of quails treated with low and high doses of Ag-NPs and AgNO_3_.

Parameter	Control	Ag-NPs (10 mg/kg)	Ag-NPs (20 mg/kg)	AgNO_3_ (10 mg/kg)	AgNO_3_ (20 mg/kg)	*p* Value
Shell weight (g)	0.85 ± 0.03 ^b^	1.54 ± 0.55 ^a^	0.93 ± 0.08 ^b^	0.83 ± 0.13 ^b^	0.79 ± 0.47 ^c^	0.022 *
Shell thickness(mm)	0.17 ± 0.01	0.51 ± 0.58	0.18 ± 0.00	0.17 ± 0.01	0.18 ± 0.02	0.434 ^NS^
Albumen weight (g)	4.61 ± 0.24	6.03 ± 0.07	5.29 ± 0.60	4.84 ± 1.21	5.60 ± 0.11	0.103 ^NS^
Yolk weight (g)	3.66 ± 0.27 ^b^	4.85 ± 0.07 ^a^	4.08 ± 0.09 ^a^	3.00 ± 0.48 ^b^	2.75 ± 0.03 ^c^	0.001 **
Haugh Unit	96.57 ± 1.0 ^b^	100.66 ± 1.4 ^a^	98.31 ± 5.65 ^b^	92.57 ± 1.90 ^c^	89.43 ± 4.74 ^c^	0.043 *
Shape Index (%)	77.41 ± 2.29 ^b^	80.70 ± 0.55 ^a^	79.38 ± 0.83 ^a^	74.89 ± 2.43 ^c^	72.92 ± 2.52 ^c^	0.006 **
Yolk Index (%)	46.85 ± 0.92 ^b^	49.09 ± 0.61 ^a^	47.65 ± 1.15 ^b^	42.09 ± 1.82 ^c^	41.06 ± 2.07 ^c^	0.003 **

Ag-NPs = Silver nanoparticles, AgNO_3_ = Silver nitrate: The mean values with distinct superscripts (^a, b, c^) in a row exhibit a substantial variation at (*p* < 0.05). ^NS^ = Non-Significant. * *p* < 0.05; ** *p* < 0.01.

**Table 5:** Reproductive Performance (Mean ± SD) of *C. japonica* treated with low and high doses of Ag-NPs and AgNO_3_.

Parameter	Control	Ag-NPs (10 mg/kg)	Ag-NPs (20 mg/kg)	AgNO_3_ (10 mg/kg)	AgNO_3_ (20 mg/kg)	*p* Value
Fertility %	79.35 ± 0.33 ^c^	81.50 ± 0.13 ^a^	80.00 ± 0.19 ^b^	77.27 ± 0.27 ^d^	76.00 ± 0.24 ^e^	0.001 ***
Hatchability %	78.05 ± 0.22 ^c^	83.28 ± 0.29 ^a^	80.91 ± 0.59 ^b^	75.00 ± 0.52 ^d^	73.00 ± 0.51 ^e^	0.001 ***

SD = Standard deviation, Ag-NPs = Silver nanoparticles, AgNO_3_ = Silver nitrate: The means values with distinct superscripts (^a, b, c, d, e^) in a row exhibit a substantial variation at (*p* < 0.05). *** = Highly significant.

**Table 6:** Bioaccumulation in reproductive organs (Mean ± SD, ug/g) of *C. japonica* treated with low and high doses of Ag-NPs and AgNO_3_.

Parameter	Control	Ag-NPs (10 mg/kg)	Ag-NPs (20 mg/kg)	AgNO_3_ (10 mg/kg)	AgNO_3_ (20 mg/kg)	*p* Value
Ovary	0.17 ± 0.03 ^d^	0.70 ± 0.04 ^b^	0.97 ± 0.05 ^a^	0.51 ± 0.05 ^c^	0.69 ± 0.06 ^b^	0.001 ***
Testes	0.13 ± 0.03 ^d^	2.08 ± 0.03 ^b^	3.38 ± 0.13 ^a^	0.24 ± 0.03 ^c^	0.45 ± 0.03 ^c^	0.001 ***

SD = Standard deviation, Ag-NPs = Silver nanoparticles, AgNO_3_ = Silver nitrate: The means values with distinct superscripts (^a, b, c, d^) in a row exhibit a substantial variation at (*p* < 0.05). *** = Highly significant.

**Table 7:** Regression analysis between bioaccumulation of Ag in ovaries and fertility (Mean ± SD) of *C. japonica* treated with low and high doses of Ag-NPs and AgNO_3._

Parameter	Control	Ag-NPs (10 mg/kg)	Ag-NPs (20 mg/kg)	AgNO_3_ (10 mg/kg)	AgNO_3_ (20 mg/kg)	*p* Value
Ovary	0.17 ± 0.03 ^d^	0.70 ± 0.04 ^b^	0.97 ± 0.05 ^a^	0.51 ± 0.05 ^c^	0.69 ± 0.06 ^b^	0.314
Fertility %	79.35 ± 0.33 ^c^	81.50 ± 0.13 ^a^	80.00 ± 0.19 ^b^	77.27 ± 0.27 ^d^	76.00 ± 0.24 ^e^	
Correlation (r)	0.136					
Regression R^2^	0.019 (1.9%)					
Adjusted R^2^	−0.057					
Slope (B)	1.10					
Slope *p*-value	0.628					

SD = Standard deviation, Ag-NPs = Silver nanoparticles, AgNO_3_ = Silver nitrate: The means values with distinct superscripts (^a, b, c, d, e^) in a row exhibit a substantial variation at (*p* < 0.05).

**Table 8:** Regression analysis between bioaccumulation of Ag in ovaries and hatchability (Mean ± SD) of *C. japonica* treated with low and high doses of Ag-NPs and AgNO_3_.

Parameter	Control	Ag-NPs (10 mg/kg)	Ag-NPs (20 mg/kg)	AgNO_3_ (10 mg/kg)	AgNO_3_ (20 mg/kg)	*p* Value
Ovary	0.17 ± 0.03 ^d^	0.70 ± 0.04 ^b^	0.97 ± 0.05 ^a^	0.51 ± 0.05 ^c^	0.69 ± 0.06 ^b^	0.153
Hatchability %	78.05 ± 0.22 ^c^	83.28 ± 0.29 ^a^	80.91 ± 0.59 ^b^	75.00 ± 0.52 ^d^	73.00 ± 0.51 ^e^	
Correlation (r)	0.283					
Regression R^2^	0.080 (8.0%)					
Adjusted R^2^	0.010					
Slope (B)	4.12					
Slope *p*-value	0.306					

SD = Standard deviation, Ag-NPs = Silver nanoparticles, AgNO_3_ = Silver nitrate: The means values with distinct superscripts (^a, b, c, d, e^) in a row exhibit a substantial variation at (*p* < 0.05).

## Data Availability

Data is available within the manuscript.
